# Supplementary material for: Changes in the salt content of packaged foods sold in supermarkets between 2015–2020 in the United Kingdom: A repeated cross-sectional study
Source: PLoS Med. 2022 Oct 5;19(10):e1004114. doi: 10.1371/journal.pmed.1004114 (PMC9581353; doi:10.1371/journal.pmed.1004114)
Supplement: S1 Table — (PDF) [file pmed.1004114.s002.pdf]

**S1 Table: Number of brands and products included in the study by year and category**

| Category | 2015 | | | | 2016 | | | | 2017 | | | | 2018 | | | | 2019 | | | | 2020 | | | |
|---|---|---|---|---|---|---|---|---|---|---|---|---|---|---|---|---|---|---|---|---|---|---|---|---|
| | Number of brands | Total number of products | Products matched with brands | Products matched with 'others' | Number of brands | Total number of products | Products matched with brands | Products matched with 'others' | Number of brands | Total number of products | Products matched with brands | Products matched with 'others' | Number of brands | Total number of products | Products matched with brands | Products matched with 'others' | Number of brands | Total number of products | Products matched with brands | Products matched with 'others' | Number of brands | Total number of products | Products matched with brands | Products matched with 'others' |
| Bread | 18 | 1091 | 809 | 282 | 18 | 1112 | 822 | 290 | 18 | 1197 | 876 | 321 | 18 | 1753 | 1297 | 456 | 18 | 685 | 552 | 133 | 18 | 777 | 574 | 203 |
| Breakfast cereals | 41 | 782 | 571 | 211 | 38 | 762 | 532 | 230 | 40 | 759 | 531 | 228 | 38 | 1262 | 900 | 362 | 38 | 635 | 431 | 204 | 38 | 666 | 446 | 220 |
| Butter and spreads | 21 | 254 | 167 | 87 | 20 | 275 | 174 | 101 | 22 | 296 | 184 | 112 | 21 | 401 | 272 | 129 | 21 | 210 | 167 | 43 | 20 | 229 | 192 | 37 |
| Cheese | 29 | 1181 | 820 | 361 | 29 | 1134 | 789 | 345 | 29 | 1159 | 813 | 346 | 31 | 1820 | 1295 | 525 | 35 | 1023 | 740 | 283 | 35 | 985 | 644 | 341 |
| Meat, seafood and alternatives | 62 | 3495 | 2712 | 783 | 61 | 3690 | 2800 | 890 | 64 | 3669 | 2772 | 897 | 64 | 6006 | 4420 | 1586 | 60 | 3068 | 2303 | 765 | 61 | 3054 | 2160 | 894 |
| Processed beans, potatoes and vegetables | 32 | 986 | 666 | 320 | 31 | 1039 | 706 | 333 | 31 | 1052 | 702 | 350 | 33 | 1508 | 985 | 523 | 35 | 642 | 453 | 189 | 35 | 665 | 438 | 227 |
| Ready meals, soup and pizza | 53 | 3361 | 2382 | 979 | 54 | 3403 | 2306 | 1097 | 52 | 3669 | 2607 | 1062 | 57 | 5546 | 3798 | 1748 | 56 | 3518 | 2628 | 890 | 57 | 3363 | 2371 | 992 |
| Sauces, gravy and condiments | 79 | 1387 | 680 | 707 | 74 | 1322 | 638 | 684 | 74 | 1362 | 647 | 715 | 77 | 2724 | 1080 | 1644 | 77 | 1416 | 608 | 808 | 73 | 1566 | 634 | 932 |
| Savoury snacks | 58 | 1103 | 685 | 418 | 58 | 1187 | 709 | 478 | 56 | 1188 | 672 | 516 | 60 | 1896 | 948 | 948 | 56 | 1416 | 1005 | 411 | 57 | 1566 | 1173 | 393 |
| **Total** | **393** | **13640** | **9492** | **4148** | **383** | **13924** | **9476** | **4448** | **386** | **14207** | **9660** | **4547** | **399** | **22916** | **14995** | **7921** | **396** | **12033** | **8307** | **3726** | **394** | **12166** | **7927** | **4239** |
