# Supplementary material for: Changes in the salt content of packaged foods sold in supermarkets between 2015–2020 in the United Kingdom: A repeated cross-sectional study
Source: PLoS Med. 2022 Oct 5;19(10):e1004114. doi: 10.1371/journal.pmed.1004114 (PMC9581353; doi:10.1371/journal.pmed.1004114)
Supplement: S2 Table — (PDF) [file pmed.1004114.s003.pdf]

S2 Table: Sales-weighted mean salt content (g/100g) by data source and category for 2018

**Category**	**Brand View (g/100g)**	**foodDB (g/100g)**	**Wilcoxon Rank Sum test (p-value)**
Bread	0.92	0.92	0.58
Breakfast cereals	0.45	0.40	0.39
Butter and spreads	1.14	1.07	0.3
Cheese	1.57	1.57	0.46
Meat, seafood and alternatives	1.39	1.30	0.28
Processed beans, potatoes and vegetables	0.58	0.42	0.15
Ready meals, pizza and soup	0.64	0.72	0.27
Sauces, gravy and condiments	1.51	1.47	0.82
Savoury snacks	1.69	1.59	0.43
**Total**	**0.96**	**0.99**	**0.4**
