# Supplementary material for: Changes in the salt content of packaged foods sold in supermarkets between 2015–2020 in the United Kingdom: A repeated cross-sectional study
Source: PLoS Med. 2022 Oct 5;19(10):e1004114. doi: 10.1371/journal.pmed.1004114 (PMC9581353; doi:10.1371/journal.pmed.1004114)
Supplement: S3 Table — (PDF) [file pmed.1004114.s004.pdf]

S3 Table: Results of sensitivity analysis - sales-weighted mean salt content (g/100g) by category and year, with 'others' removed

**Grouped category**	**2015**	**2016**	**2017**	**2018**	**2019**	**2020**	**Percentage change 2015-2020 (%)**	**Absolute change 2015-2020 (g/100g)**	**Kruskal-Wallis test (p-value)**
Bread	0.93	0.92	0.93	0.91	0.92	0.92	-0.97	-0.01	0.56
Breakfast cereals	0.51	0.49	0.46	0.45	0.41	0.42	-17.76	-0.09	0.39
Butter and spreads	1.17	1.20	1.14	1.11	1.10	1.14	-2.49	-0.03	0.30
Cheese	1.67	1.66	1.64	1.57	1.63	1.62	-3.15	-0.05	0.46
Meat, seafood and alternatives	1.45	1.43	1.39	1.29	1.29	1.27	-12.27	-0.18	0.28
Processed beans, potatoes and vegetables	0.54	0.53	0.53	0.49	0.42	0.44	-17.64	-0.10	0.15
Ready meals, soup and pizza	0.68	0.65	0.65	0.64	0.65	0.64	-5.51	-0.04	0.26
Sauces, gravy and condiments	1.54	1.54	1.53	1.56	1.51	1.47	-4.86	-0.07	0.81
Savoury snacks	1.74	1.74	1.70	1.62	1.60	1.58	-9.01	-0.16	0.43
**Total**	**1.01**	**1.00**	**0.99**	**0.96**	**0.95**	**0.95**	**-6.23**	**-0.06**	**0.40**
