# Supplementary material for: Changes in the salt content of packaged foods sold in supermarkets between 2015–2020 in the United Kingdom: A repeated cross-sectional study
Source: PLoS Med. 2022 Oct 5;19(10):e1004114. doi: 10.1371/journal.pmed.1004114 (PMC9581353; doi:10.1371/journal.pmed.1004114)
Supplement: Attachment — Submitted filename: response_to_reviewers_letter.docx [file pmed.1004114.s005.docx]

**Response to reviewers letter**

Thank you to the editors and reviewers for taking the time to review this manuscript. We have taken your feedback on board, especially with regards to the concerns around the validation between our two composition datasets, and the impact the sales values for the “others” category has had on our results. We have conducted a validation exercise and a sensitivity analysis to address this, which are presented in the manuscript. During this process, we also spotted an error in our code for how “others” were being calculated and incorporated into our dataset, and have re-run the analysis and updated the figures and results accordingly, finding a slightly smaller (and still statistically insignificant) change in the sales-weighted mean salt content of foods over time.

We have responded to each comment below, including line numbers of the clean manuscript.

Requests from the editors:

Please adapt the title to better match journal style. We suggest: "Changes in the salt content of packaged foods sold in the United Kingdom during 2015-2020: A repeated cross-sectional study".

The title has been updated as suggested.

Where available, please quote p values in the abstract.

These have been added

Please add a new final sentence to the "Methods and findings" subsection of your abstract, which should begin "Study limitations include ..." and should quote 2-3 of the study's main limitations.

This has been done and lines 33-36 now read: *“This study is limited by its exclusion of foods purchased out of the home at restaurants, cafes and takeaways. It also does not cover salt added at the table, or that naturally occurring in foods, meaning the findings are likely to underrepresent the population’s salt intake.”*

It is not clear that the sentence beginning at line 35 is a "conclusion", and we ask you either to remove this or to add "We suggest that ..." or similar.

This has been updated as suggested

After the abstract, please add a new and accessible "author summary" section in non-identical style. You may find it helpful to consult one or two recent research papers published in PLOS Medicine to get a sense of the preferred style.

This has been added

At line 82, please remove "fellowship project".

This has been updated as suggested here and by reviewer 3.

Around line 88, please check that "Ascential" is spelt consistently.

Thanks, typo corrected.

Early in the Methods section (main text), please state whether the study had a protocol or prespecified analysis plan and if so attach the relevant document as an attachment, referred to in the text.

This has been updated as suggested.

Please highlight analyses that were not prespecified

This has been done.

Noting the p values in table 1, for example, please note the statistical test used in a legend.

Column headers for Table 1 have been updated

Throughout the text, please amend reference call-outs to the following style: "... blood pressure [4,5]." (noting the absence of spaces within the square brackets).

In the reference list, we suggest spelling out institutional author names such as "Strategy NF".

Please include URLs for reports such as reference 10, where available, along with accessed dates.

Referencing style has been updated to PLOS Medicine and hopefully these formatting issues should now be resolved.

Please add a completed checklist for the most appropriate reporting guideline, e.g., STROBE, as an attachment, labelled "S1_STROBE_Checklist" or similar and referred to as such in the Methods section (main text).

In the checklist, please refer to individual items by section (e.g., "Methods") and paragraph number, not by line or page numbers as these generally change in the event of publication.

STROBE checklist uploaded and labelled as requested

*** Reviewer #1:

I confine my remarks to statistical aspects of this paper. The general approach is fine, but I have some issues and suggestions before I can recommend publication.

p. 3 line 40 - I'm guessing this is per year, but you should state that.

Thanks, this was unclear and has been corrected.

Line 69 - similarly, I'm guessing this is per day, but you should say

This has been updated and lines 88-92 now read*: “A study that analysed the sodium content of 47,000 foods purchased in 18,000 households in the UK between 2006 and 2011 found that the mean content fell by 7% overall, concluding that the voluntary reduction targets had delivered a moderate reduction in the salt content of foods over the time period studied (11).”*

p. 4 lines 86-94 Maybe put in a sentence such as "the method of reconciling these sources is discussed on p. 6" or something like that.

The methods section has been updated after various comments and we hope this is now clearer.

Table 1 - a minor point, and maybe I am wrong, but I'd consider ordering this table by %, rather than alphabetically. But I can also see reasons to leave it as is.

We agree with the reviewer and have reordered it by % rather than alphabetically.

p. 7 line 155-6 I think you need to give the formula here, but I leave that to the editors' judgment.

Thanks for your comment – we felt it was a relatively simple calculation and as the editor has not suggested a formula, we will leave it as it is unless advised otherwise.

Fig 3, 4 - what do the black segments represent? Gray is sales, orange is salt. (On examination, kit looks like the black line is the total change. But 1) You should say this and 2) the black should be narrower, like a line rather than a box.

Thanks, we have made this clearer by adding a description of the black marker into the key and explaining the figure in the text as suggested.

*** Reviewer #2:

The topic of this paper is noteworthy, which investigates the salt content of packaged foods sold in the UK between 2015 and 2020. The authors report that there has been a steady decline in the salt content of packaged foods and total volume of salt sold between 2015 and 2020, with considerable differences between categories and companies. However, I have some concerns and suggestions for the authors, which I have detailed below.

Major comments:

1.The unit of analysis is the brand and the sales-weighted mean salt content per brand is the main primary outcome. There is a range of product for each brand. Within each brand, different sale quotas might be allocated to different products. Is it possible to segregate product sales? If such data is available, it would be better to explain why brand-level data should be used and to conduct sensitivity analysis (e.g. analyse the impact of individual product variation within each brand on the overall sales-weighted mean salt content of foods).

It is not possible to differentiate between individual product sales under the same brand with Euromonitor data and we recognise that this is a major limitation. We have expanded on this in the discussion, and lines 353 to 363 now read: *“The advantages and limitations of using Euromonitor sales data to analyse the nutrient content of foods over time have been outlined in detail in a previous study (17). In summary, Euromonitor sales data avoids reliance on individual recall and underreporting in scan data (24), and it also allows for analysis of individual brands and companies. However, its limitations mean that table salt and salt sold from fast food are both excluded, making the total volume of salt sold likely to be a significant underestimation, and the Euromonitor’s reporting of brand sales, and not individual product sales, means that any heterogeneity that occurred between products that belong to the same brand would have been missed, given that the method used assumes all products under the same brand are sold equally. A previous study has examined this limitation in detail, concluding that while the overall findings are unlikely to be affected, there may be some significant misrepresentations at the brand and potentially company level.”*

2.Page 7: dietary recommendations for adults and children are different. Total volume of salt sold in grams per person per day in this paper is averaged throughout the whole population, including adults and children. Meanwhile, the method used to determine it cannot be compared with 24h urine estimates. Therefore, more discussion is needed.

Thank you, lines 348-351 of the discussion now read: *“It is also important to note that the sales data do not provide any split by demographics and the per person per day estimates presented here are relatively crude and include both adults and children. They are therefore not directly comparable to the results of the urinary sodium figures, which are for adults only.”*

3.Page 6: small and local brands are grouped under the umbrella term "others" and the remaining 40,003 products in the composition database are assigned to "others" within each category. It is unclear whether the sales volume data and nutrition information for the category "others" match exactly. A sensitivity analysis is required if this is not the case.

We have added a sensitivity analysis that repeats the analysis with the “others” category excluded. After excluding “others”, the overall sales-weighted mean salt content of foods was 0.97g/100g in 2020, compared to 0.93g/100g as presented in the main findings. The sales-weighted mean salt content and trends in change over time were similar across all categories (S3 Table), with the exception of two categories. The ready meals, soup and pizza category saw salt content fall from 0.67g/100g in 2015 to 0.64/100g in 2020 (-4.5%) in the sensitivity analysis, compared to +1.0% in the main findings, and the processed beans, potatoes and vegetables category saw salt content fall from 0.54g/100g in 2015 to 0.44g/100g in 2020, a reduction of -18.6% compared to -11.5% in the main findings. This suggests that overall, the “others” category has little impact on the main findings, although there are slight differences in the trends for the processed beans and ready meal categories. This sensitivity analysis has been written up in full, and is included in the methods, results and discussion sections of the manuscript.

4.Page 8: the P-values in Table 1 for changes in sales-weighted mean salt content (g/100g) of the 9 food categories are not statistically significant. In this case, reported deduction in the salt content of selected packaged foods might be caused by a random variation. As a result, conclusions can be misleading. It is necessary to provide clarifications or amend the conclusions so that they reflect the findings obtained.

Thanks, we have added a statement to say the results were not statistically significant into the abstract, results and discussion, so as to not overstate the findings.

5.Page 8: In Table 1, changes in sales-weighted mean salt content of 9 food categories are calculated between 2015 and 2020. As the yearly annual data is available, would be better to conduct an analysis that would account for / make use of all the data available.

One of the main outcomes of this paper is to assess how industry-led changes in the salt content of foods has changed in comparison to changes in volume sales of food. To do this, we need to use the absolute figures in 2020 and 2015, using the formula that has been outlined in the methods section (lines 198-205). Calculating the average annual change would not allow us to examine the change in sales (a proxy for consumer behaviour) and the change in mean salt content (a proxy for industry behaviour).

We agree though that making use of more data would be better. We have therefore added an additional figure (figure 2), that better illustrates how the sales-weighted mean salt content has changed over time than the table does alone.

Minor comments:

1.Page 3, line 69-71: citation 13 contains information that differs from that found in the reference paper (He FJ et al., 2014. Salt reduction in England from 2003 to 2011: its relationship to blood pressure, stroke, and ischaemic heart disease mortality). Please double-check it.

Thanks for this – we’ve double checked all the references for this paragraph. It’s been updated and lines 100-108 now read: *“Assessment of salt intake from urinary sodium excretion in adults has shown that estimated mean salt intake reduced from 8.1g in 2005-6 to 7.2g in 2014by 1.4g/day between 2003-2011, contributing to a reduction of 3.0mmHg in diastolic blood pressure between 2001 and 2011 and a reduction in mortality from stroke and ischaemic heart disease (13). The most recent data from the National Diet and Nutrition Survey’s 2018-19 survey assessment of urinary sodium in adults reported a mean intake of 8.4g/day (14), with no significant change observed compared to 2014, suggesting the population’s salt intake has plateaued in more recent years, suggesting salt intakes have creeped back up more recently.”*

2.Page 5: NDNS contributors in Table 1 needs a detailed explanation.

Table 1 has been updated with clearer column headers

3.Black bars in Figure 3 (page 10) and Figure 4 (page 11) need an explanation.

Thanks, we have made this clearer by adding a description of the black marker into the key and explaining the figure in the text as suggested.

4.Page 8: The change in the sales-weighted mean salt content of bread (Table 1) between 2015 and 2020 is -0.01. When comparing the sales-weighted mean salt contents of bread in 2015 (0.93) and 2020 (0.93), the difference is 0. Therefore, it would be better to double check it and keep the right decimal.

Thanks for spotting this – the calculations were done before the rounding, hence the discretions, and we have added a footnote to highlight this.

5.It would be better to discuss the implications of excluding dried sauces, gravy, and stock cubes.

We agree that this was an oversight and lines 380-387 of the discussion now read: *“Dried sauces and gravies were excluded from the dataset as no data was given to indicate whether the salt content values were as sold or as given. After investigation, most products had their own preparation instructions and attempts to come up with a generic formula that could be used to convert all values over a certain threshold to as sold (as the sales data is given as sold) caused jumps in the category results across years, therefore it was decided to exclude dried products. While the high salt content of dried products makes them a category of interest, results from the NDNS data show that the total sauces category represents 6% of salt intake overall, and it is not expected that their exclusion will have a large impact on the overall results.”*

6. In the discussion section, would be better to mention Gressier et al. 2021's paper "Contribution of reformulation, product renewal, and changes in consumer behavior to the reduction of salt intakes in the UK population between 2008/2009 and 2016/2017".

Thank you for this suggestion – this paper is now referenced in the introduction and discussion.

7.Page 12, line 217: it would be better to clarify that the sales-weighted mean salt content of packages foods fell by 0.07g per 100g.

Thanks, this has been updated

8.Page 13, line 223, please check that the comma is in the correct position.

Thanks, this has been corrected

*** Reviewer #3:

This paper looks at the changes in salt content of foods between 2015 and 2020 in the UK. It's interesting work and relevant to understand how prior voluntary policies have managed to shape nutrient consumption (proxied through sales). I do have some comments and queries regarding the methods in particular which I think need further clarification.

-	Introduction - It would be good to have a more thorough overview of the work published investigating salt reformulation; where this paper fits in and what is the added value to the literature. I know of at least three papers that would be relevant for this study (at least for background) which are not mentioned

o	https://academic.oup.com/ajcn/article/114/3/1092/6272434?login=true

o	https://jech.bmj.com/content/73/9/881

- https://onlinelibrary.wiley.com/doi/abs/10.1111/ecca.12192

The first two studies have been included in the introduction, and the third in the discussion.

-	ln53 - clarify what you mean by the target - is it 'no more' than 6g/d?

Yes, this has been updated and line 79-80 now reads: “The United Kingdom has one of the best-known salt-reduction programmes globally, aiming to achieve a population salt intake of less than 6g/d”

-	ln82 - is it important to note in methods that it is part of a fellowship project? That seems to be more appropriate to acknowledgement section together with fellowship grant reference

As suggested by the reviewer and the editor, reference to the fellowship project has been removed.

-	How good is the match between the two data-sets on salt content of products (Edge by Ascential and foodDB)? - some validation information on this (validation) would have been useful to ensure that any changes observed are not due to using two different sources for 2015 and 2020.

We have added another analysis that assessed the validation between the two sources of composition data (Edge by Ascential and foodDB) for 2018. The sales-weighted mean salt content by category was calculated for each data source, and a Wilcoxon signed-rank test was conducted in R to test for differences between the two independent samples, with the unit of analysis being the brand. When the sales-weighted mean salt content was calculated for each data source in 2018, there was no statistical difference in the results overall (p = 0.63) and by category (see S2 Table), suggesting that the results are due to genuine change in the mean salt content, rather than random variation between the two datasets. This has been detailed in the manuscript in the methods, results and discussion sections.

-	If I understand correctly there were 93,325 products matched into 395-416 brands. This means that each brand contains over 200 products and I question the use of average salt content because within such a large number of products salt content as well as popularity of individual products must vary (the very reason you do weighting at brand level) and I didn't see any clarification or justification about this.

We have added S1 Table, which gives the number of brands and products included for each year in more detail, and we hope this clarifies things.

-	Number of brands per food groups should be indicated in table 1. Table 1 should also have clearer indication to show what the last two column headings mean (i.e. % change between what, p-value for what?). Furthermore, you should use more than two decimals as two decimals show no change in salt content of bread for example.

Please see S1 Table for more detail on the number of products and brands included. The headers of Table 2 have been updated to make the % change 2015-2020 and Kruskel-Wallis test p-values clearer, as suggested.

-	Line 179 - what do you mean by meaningful?

Agree this is word has little meaning and have removed it

-	Fig 3 & 4 - what do the solid black lines represent?

Thanks, we have made this clearer by adding a description of the black marker into the key and explaining the figure in the text as suggested.

-	Given that you have the whole series between 2015 and 2020 you could have also commented on the in-between years

As mentioned in response to another reviewer’s comments, we calculated percentage change between 2015 and 2020 – rather than average annual percentage change over the full six year period – so that we could split this difference between a change in sales (proxy for consumer behaviour) and change in mean salt content (proxy for industry behaviour). This is explained in the methods section (lines 200-204): *“The change in total volume of salt sold was split into the change in the mean salt content (an indicator of reformulation) and change in total volume sales using a decomposition formula described elsewhere (17, 19). In summary, the annual percentage change in the total volume of salt sold is equal to the sum of the annual percentage change in the mean salt content and the annual percentage change in the volume of foods sold. This was calculated at the total, category and company level.”*

We have added a new figure, Fig.2, that gives a better demonstration of change in the in between years than the table does alone.

-	The numbers in figure 3 does not match to the text explaining figure 3 so it makes a confusing read

The first lines (254-258) of the text explaining figure 3 now read: “*The total volume of salt sold fell by 7.6% between 2015 and 2020 (represented by the black marker in Fig.4 below). The majority, or 63%, of this change was due to reductions in the mean salt content of brands (represented by the orange bar in Fig.4), with the remaining 37% due to a decline in the volume sales of products (represented by the grey bars in Fig.4).”* We hope this makes the explanation clearer.

-	Table 1 shows that the decline in salt content was not significant at conventional statistical significance level and start the discussion states the decline as if it was. If you have chosen to ignore this for some reason, this should be carefully explained and why can you can still conclude this finding.

We recognise this, and in the abstract, results, discussion/conclusion and abstract, have highlighted that the decline was not statistically significant as not to overstate the findings.

*** Reviewer #4:

Bandy et al reported the changes in salt sold in the UK through retail between 2015-2020.

While the manuscript is generally well-written and the study well-conducted, there are several major issues which I would like to invite the authors to address:

1. A main limitation of the current study is the reliance of data collected from websites. There is no information provided in the manuscript, as to how the research team had assessed the accuracy of the data.

A validation study using the foodDB database comparing products collected online with those in store has already been done and added to the discussion (lines 372-374). In summary, while online grocery shopping is still small compared to in-store sales, the availability, price and nutrition information of products collected online are all comparable to those in-store and we therefore do not expect this to be a major limitation.

2. The authors selected nine categories, and claimed that eight of these represented ~73% of an adult's dietary sodium intake from processed foods. However products purchased are not only consumed by adults, but also children and adolescents.

We understand that these foods are consumed by both children and adults, and have clarified in the table header that the percentages given are for adults (19-65 years).

3. Line 126: What is defined by Brand? e.g. For Kellogg's Special K, is Kellogg's or Special-K the Brand? This distinction is important given the sales data are weighted by "Brand", and if in the example above "Kellogg's" is identified as the Brand then the accuracy of the sales-weighted estimation will be too crude. This needs to be clarified. It is also surprising that only ~400 Brand exist in the UK market - does that mean each brand represent ~350 products (136k/395)?

We have added an example in the methods. Lines 166-172 now read: *“Euromonitor measures sales by brand and so individual product sales data were not available. A brand was defined as a set of products that have the same core name and are manufactured by the same company. For example, the company Kellogg’s manufactures multiple brands, including Special K (one brand) and Coco Pops (another brand) breakfast cereals. Within each brand, there may be multiple individual products (e.g. Special K Original and Special K Red Berries).”*

We have also added S1 Table, which gives a break-down of how many products and brands were included for each year.

4. Another issue is that the current study only examined packaged foods sold in the supermarket, as the authors themselves acknowledged.

We have made an extra note in the discussion to highlight that this study is limited to only packaged grocery foods, and excludes foods consumed out of the home. Lines 374-378 now read: *“It is important to note however that the data used in this study does not represent all grocery foods, and also excludes products sold out of the home in restaurants, cafes and canteens for example. Further research in this sector is warranted in the future, given that food consumed out of the home represents an estimated 24-39% of total food spend {Cornelsen, 2019 #3523}.”*

5. The discussion is very brief. The authors should consider expanding it by including some or all of the following:

i) likely reasons for the small change observed, e.g. reluctance of the food industry, lack of consumer acceptance for lower Na products, technological challenges, etc.

ii) proportion of products meeting target over time

iii) likely impact of the current progress on diseases reduction (or lack thereof)

iv) strategies to enhance the compliance to the targets

v) complementary strategies for salt intake reduction in the population

We recognise that the discussion is concise, but we feel it is comprehensive. Due to limitations in the sales data, we are not able to compare our results directly with the very specific categories of the salt reduction targets, although we plan to do work in this area in the future (as discussed in lines 374-378). We also suggest that mandatory reporting of salt sales, and reporting of data to common standards would allow policy makers, academics and others to better track companies’ progress towards the targets (lines 400-404), and that policies that encompass consumer behaviour change are also needed alongside industry-led targets (line 395-398). We are planning on doing some modelling work on the health impact of current progress towards the salt reduction targets in the future, but feel it is beyond the scope of this paper to speculate on disease reduction.

There are also some minor issues that should be addressed:

1. Line 53: should this be < 6 g/day?

This has been corrected

2. Line 112: do you mean savory or sweet biscuits? Also most of these quoted items are not low in sodium (as defined by the FSA traffic light label cut-offs), but only relatively lower in sodium. Excluding them may introduce error especially underestimation given some of these items were also included in the reformulation targets as far as I understand. "Making the categorization process more manageable" does not seem like a sound justification to me.

This has been updated to make it clear that is sweet biscuits that were excluded (line 153). We have added more detail into the methods with regards to the categorisation progress (lines 145-148), but given the manual process and limited resources available, we feel that prioritising high-salt categories (as laid out in table 1) was justified.

3. Regarding dried sauces/gravy etc. there could be some ground rules that would allow their inclusion. e.g. the difference between the dried vs made up version of these products would have huge differences in the sodium level, although this may require some manual checking.

We did try to come up with a generic formula to identify which dried sauces gave their salt content values as consumed, as opposed to as sold. However, given the number of products and the wide range of salt contents, there was no easy g/100g threshold that we could identify and applying a generic formula caused jumps in the data over time for this category. We recognise that this is a limitation and not fully explained, therefore we have added the following paragraph to the discussion (lines 380-388):

*“Dried sauces and gravies were excluded from the dataset as no data was given to indicate whether the salt content values were as sold or as given. After investigation, most products had their own preparation instructions and attempts to devise a generic formula that could be used to convert all values over a certain threshold to as sold (as the sales data is given as sold) caused jumps in the category results across years, therefore it was decided to exclude dried products. While the high salt content of dried products makes them a category of interest, results from the NDNS data show that the total sauces category represents 6% of salt intake overall, and it is not expected that their exclusion will have a large impact on the overall results.”*

4. The use of mean salt content for variations within a Brand may introduce error as their sales volumes are different.

This is the main limitation of this paper, and has been expanded on in the discussion. Lines 358-363 now read: *“Euromonitor’s reporting of brand sales, and not individual product sales, means that any heterogeneity that occurred between products that belong to the same brand would have been missed, given that the method used assumes all products under the same brand are sold equally. A previous study has examined this limitation in detail, concluding that while the overall findings are unlikely to be affected, there may be some significant misrepresentations at the brand and potentially company level.”*

5. Line 152: presenting the data as salt purchased per day per person and compared that across the year is likely misleading, given the population make up/demographics may have changed over the years. This should be discussed as a limitation.

We found that presenting salt purchased in grams per person per day was the most useful metric when reporting the total volume of salt sold, as it puts it in context of dietary guidelines, as opposed to using tonnes. To calculate the g/day values, we used annual ONS statistics for 2015-2020 so that annual changes were incorporated. We recognise that the sales data is limited as it does not split down further by age group or any other demographic factor. We have highlighted this in the discussion and lines 348-350 now read: *“It is also important to note that the sales data do not provide any split by demographics and daily per capita estimates presented here are relatively crude and include both adults and children. They are therefore not directly comparable to the results of the urinary sodium figures.”*

6. No justification was provide for the use of the non-parametric Kruskal-Wallis test over its parametric counterpart. Was the normality of the data examined? If so, how? Also no p value cut-off was given.

We have updated the methods so that lines 205-207 now read: *“As the data were not normally distributed (assessed by visual inspection), differences in the sales-weighted mean salt content of each category in 2020 compared to 2015 were tested using a Kruskal-Wallis test, where brands (rather than sales) were used as the unit of analysis.”*

7. Line 187: describing items which are no longer available for sale as "due to decrease in sales" is inaccurate.

We agree items that are no longer available does not mean a decrease in sales. We cannot find where this was implied.

8. Figure 3: No description on what the black bar means is given.

Thanks, we have made this clearer by adding a description of the black marker into the key and explaining the figure in the text as suggested.
