# Supplementary material for: Changes in the salt content of packaged foods sold in supermarkets between 2015–2020 in the United Kingdom: A repeated cross-sectional study
Source: PLoS Med. 2022 Oct 5;19(10):e1004114. doi: 10.1371/journal.pmed.1004114 (PMC9581353; doi:10.1371/journal.pmed.1004114)
Supplement: Attachment — Submitted filename: reviewers_response_letter.docx [file pmed.1004114.s006.docx]

**Letter of response to reviewers**

We thank the editors and the reviewers for taking the time to look at our manuscript again, and are pleased to see that Reviewers 1-3 considered their comments to have been addressed at the last round of revisions.

We hope that by removing duplicate products found in multiple retailers we have addressed the main concerns of Reviewer 4. We recognise that using the brand as the unit of analysis is a limitation, which we have discussed fully, but do not have access to individual level products sales data and so are limited with how far we can overcome this limitation.

Requests from the editors:

The bullet at line 53 doesn’t seem to be complete.

Thanks, this sentence has been removed and the line above edited accordingly

S1 and S2 Tables are duplicated, and the STROBE checklist is missing.

Apologies for the mix up, these are correct now

Table S3 column headers appear to be truncated.

This has been updated

Line 116: Please state in this subsection that your study does not have an associated protocol.

This has been updated

Line 148: Please include the software manufacturers.

This has been done

Line 323: Please confirm this remains accurate and add ‘to our knowledge’.

We can confirm this is still true, and have added ‘to our knowledge’.

Lines 389-390: Please update to ‘This analysis suggests that salt intake from processed foods purchased in retail settings fell by 6.9% between 2015-2020, but this decrease was not statistically significant.’

This has been updated

Please include the date accessed for references 10 and 31.

For some reason Endnote would not add these, so I’ve added them manually

Comments from the reviewers:

Reviewer #1: The authors have addressed my concerns and I now recommend publication.

Peter Flom

Reviewer #2: Overall, all of the comments have been addressed by the authors. I still doubt the validity of the datasets, e.g. duplicate counting of products sold in multiple retailers, which could affect the results. I leave that to the editors' judgment. A minor comment is that reference 14 should be checked properly. Instead of diastolic blood pressure, systolic blood pressure fell by 3.0mmHg.

Thanks, we have double checked the reference as recommended and have updated the blood pressure to systolic. We have also removed duplicate products from multiple retailers and re-run the analyses (taking the total number of including products across all 6 years from 136,000 to 88,000), finding only small differences to the results which have been updated. We hope this goes some way to addressing the reviewer’s concerns regarding data validity.

Reviewer #3: All my comments have been responded to. I only have few minor comments to further make

Abstract

Some p-values missing

Can you provide p-values also for the change in total volume of salt (also in results section?)

Thanks, we have added the missing p-values as suggested above

Author summary

Check the first bullet point in the second section - it does not seem to be a complete sentence

Last bullet point - this also needs a clarification that the biggest reductions were not statistically significant either

Thanks, we have edited both of these bullet points as suggested above

Results

The numbers cited in line 231-233 seem to be in reverse with reference to the year they are from. Sentences are missing the word 'products'

Thanks, we have corrected this.

Discussion

First sentence - the p-value should be 0.23?

p-values have been updated throughout

Linde 355 - what do you mean by 'industry behaviour' beyond reformulation as that is already mentioned. Consumer behaviour could also be explained a bit better- is it both buying less and buying products with lower salt content?

Thanks for your comment, we agree that it is best to be explicit by actions included under both industry and consumer behaviour changes. Lines 410-415 now read:

“*This highlights the need for policies that encompass consumer behaviour change and purchasing (such as reducing consumption of high-salt products and shifting towards low-salt varieties) as well as industry-led salt reduction targets and the launch of new low-salt varieties. The salt reduction programme in the UK was initially credited with such success because it included a public awareness campaign and strong political support [32], both of which have waned in recent years.”*

S3 table - column and row headings are not fully visible

S4 table - add also the Kruskal Wallis test as in table 2

Thanks, all the supplementary files have been corrected

Reviewer #4: I thank the authors for their attempt to address my concerns about their manuscript. I am confining my comments below to their responses to my original comments (Comment# corresponds to that in the original review)

Major:

1. What are the ~15% of offline store items not found on the website?

We assume this is in reference to the validation study [30] that found 85% of products found online were also available in store (lines 382-385). We have gone back to the paper but no further explanation is given regarding the details of the remaining 15% of products and so cannot elaborate further here.

2. No further comment.
3. I felt that the authors did not adequately address this concern (raised by 3 reviewers). In Table S1, for example, there were 18 brands of bread in 2015, but that represents 1520 products. That means in the Euromonitor dataset there were ~90-100 items in each brand. The same is true for breakfast cereals and other categories. Using the authors' own argument in the response letter, that means Kellogg's Special K as a brand represent ~30 items in 2015? That does not seem to make sense. Even if this is true, it is reasonable to suspect that these items will be highly heterogenous, and thus using "brand" as a unit of analysis is quite likely to introduce sizeable errors in the estimation of sodium purchased.

As we did not have any unique identifiers (such as barcode or SKU number) in the dataset to remove duplicate products, we left products found in multiple retailers in originally. While this might be suitable when measuring availability, we recognise that it is a limitation as outlined in the comment above. We have therefore removed duplicate products found in multiple retailers from the dataset based on year, brand name, category name, product size, kcal/100g and salt/100g. This has reduced the dataset from 136,328 to 88,886 products total across all six years. We have re-run all the analysis and found that this has had very little on the results, but all tables, figures and text have been updated accordingly. Now, for example, as outlined by S2 Table, the average breakfast cereal brand is now paired with 11 products, which seems entirely plausible given the range of product flavours and sizes available. We would also like to note that this limitation has been discussed at lengths in the discussion of this paper and that of another using the same database, which included a sensitivity analysis. Lines 366-372 read:

*“Euromonitor’s reporting of brand sales, and not individual product sales, means that any heterogeneity that occurred between products that belong to the same brand would have been missed, given that the method used assumes all products under the same brand are sold equally. A previous study has examined this limitation in detail, concluding that while the overall findings are unlikely to be affected, there may be some significant misrepresentations at the brand and potentially company level [20].”*

The paper makes it clear that using brand sales is a limitation, but we do not have access to individual-level sales data. We hope that by removing duplicates and updating the paper, we have addressed the reviewers concerns.

4. How about convenience stores? That in my opinion would be a prime source of high sodium packaged foods.

We recognised that convenience stores are likely to be a source of high-sodium foods, although suspect many of the big brands sold in convenience stores will also be sold in the four supermarkets included here. We have added the word ‘supermarkets’ to the title to make it clear from the start that this study is limited to large retailers only. We do not have access to any composition data for products sold exclusively at convenience stores, but have updated the discussion to make this limitation clearer (lines 385-388):

*“It is important to note however that the data used in this study was collected from four major retail chains only so does not represent all grocery foods, including those sold at convenience stores and markets, and also excludes products sold out of the home in restaurants, cafes and canteens for example.”*

5. I respectfully disagree with the authors' response. The current discussion is in my opinion inadequate and does not do justice to the data available, and I have in my original review provided some ideas of how the authors could better discuss their findings.

Please see the response to each of the reviewer’s original comments regarding the discussion. This response is not dissimilar to the first, and so I suspect the editor will need to make a call here as to whether or not the discussion is insufficient as the authors feel it is already comprehensive.

i) likely reasons for the small change observed, e.g. reluctance of the food industry, lack of consumer acceptance for lower Na products, technological challenges, etc.

The discussion has been updated and lines 405-408 now read: “*It is likely this is due to a combination of reasons: Many in the food industry cite the technical challenges to salt reduction, especially in products where it also acts as a preservative; there can be consumer resistance to low salt varieties, although this can be overcome by gradual salt reduction; and there has been a shift in the efforts of both industry and policy makers towards sugar and calorie reduction instead.”*

ii) proportion of products meeting target over time and iv) strategies to enhance the compliance to the targets

This study is not a specific evaluation of the salt reduction targets. The sales data we have used here does is not granular enough to align with the specific salt reduction target categories and we therefore cannot comment on compliance and what proportion of products are meeting the targets over time. We recognise that this is a limitation, as discussed in lines 375-379: “*The lack of granularity in the sales data also means that these results are not to directly comparable with PHE’s salt reduction targets, which have very narrow categories, therefore this study is not a specific evaluation of the salt reduction targets. However, the authors do have future research in this area planned, including modelling the targets’ potential health impact.”*

Lines 328-335 also read: “*However, it is hard to make any direct comparisons between these reports and this study’s findings, as the PHE salt reduction target categories are so specific it is not possible to align categories. For example, in this study, we presented results for the ‘cheese’ category, although data is available for hard cheese, processed cheese and soft cheese. The PHE targets for cheese include ‘soft white cheese’, ‘cottage cheese’, ‘mozzarella’, ‘blue cheese produced in the UK only’, ‘cheese spreads’ and ‘other processed cheese such as sliced and string cheese with emulsifiers’ [11]. The sales data we used in this study was not granular enough to calculate the sales-weighted mean salt content of these specific PHE categories.”*

iii) likely impact of the current progress on diseases reduction (or lack thereof)

We have have added the following lines to the discussion and hope this addresses the reviewers concerns.

*“Results of a meta-analysis suggest that a 4.4g/day reduction in salt intake leads to a 4.4mm Hg fall in systolic blood pressure [6]. The change in total volume of salt sold observed in this study was -0.16g/day; while any decline in salt intake is welcome, it seems unlikely that this change would have led to any substantial reductions in disease prevalence and mortality.”*

v) complementary strategies for salt intake reduction in the population

Lines 418-425 read: “The specificity of the salt reduction targets means it is very hard to assess progress towards the targets using available data sources. Mandatory reporting of data to common standards would allow policymakers, academics, investors and others to better track companies’ progress towards public health goals, as has been called for in the National Food Strategy [33]. It would shine a spotlight on parts of the food system which were leading or lagging behind policy targets, allow for a better understanding of how effective the current targets are at improving public health outcomes, and allow alternative measures to be considered, such as mandated maximum salt targets (as in Argentina) and taxes on high-salt foods (as in Portugal) [34].”

Minor:

3. I disagree that it is not easy to create a ground rule to identify dried vs. as prepared. If nutrition information panel data are available, since the overall weight equal sum of CHO + protein + fat + fibre + water + (minerals), it would be quite easy to deduce the water content (and thus whether the nutrition label presents the dried vs. as prepared data).

The authors still think it is best to exclude this category as the data is so unreliable. It is not just about identifying whether a product is as sold or as consumed, but also calculating a ratio to adjust this given the wide range of manufacturer’s preparation instructions. The results would be arbitrary and as the dry sauces category only represented 1% of volume sales of sauces (as sold) in 2020 and the sauces category 6% of total salt intake, it seems best for them to remain excluded.

4. Please see Major3 above.

5. The authors did not completely address my concern. What I meant in my original comment is that the composition of the UK population had likely changed between 2015-2020. As an arbitrary example there may be more older adults or females in 2020 cf. 2015. This would likely affect the amount sodium purchased. Using the annual ONS statistics only allows the authors to correctly calculate the per person data, but not reflect this change in population composition.

We recognise that the per person per day estimates that we have calculated here are relatively crude given that the sales data does not provide any information on demographic breakdown. According to ONS, the proportion of people aged 65+ increased from 17.8% in 2015 to 18.6% in 2020. We have edited this paragraph in the limitations section to make it clear that demographic changes are not captured in this data and lines 351-355 read:

“*It is also important to note that the sales data do not provide any split by demographics and therefore any change in population composition (e.g. increase in proportion of older people) are not captured, making the daily per capita estimates presented here are relatively crude. They include both adults and children of all ages and are therefore not directly comparable to the results of the urinary sodium figures which are for adults aged 19-64 only.”*

6. This relates to line 147-148 in the original manuscript "Change in sales-weighted mean salt content was calculated between 2015 and 2020, with all products being included regardless of whether they were present in the market in 2015 or 2020.". From the wording it is implied that the value 62% was obtained from counting both items available at both 2015 and 2020 (and had little change in sales), as well as new items introduced in 2020; while the remaining 38% were items available in both 2015 and 2020 with a decline in sales, and those only available in 2015. It is "those only available in 2015" that I was referring to as "describing items which are no longer available for sale as "due to decrease in sales" is inaccurate.". The authors should better clarify this in text if my understanding is incorrect.

We thank the reviewer for their comment and agree that this sentence in the methods is confusing. We calculated percentage change in mean salt content between 2015 and 2020. We have updated line 198 in the methods to remove the second half of the sentence – the “all products being included regardless” refers to the fact that each year the data included new products added to the market place, and excluded any products that were discontinued, i.e. this was a database of all brands and products, not just those that were present throughout the whole time series. This has been clarified in lines 179-181.

The 62% refers to the proportion of the change in total volume of salt sold that can be attributed to a change in the mean salt content, as opposed to any changes in sales volume (38%). This is explained in the methods section lines 204-211.
