# Supplementary material for: Changes in the salt content of packaged foods sold in supermarkets between 2015–2020 in the United Kingdom: A repeated cross-sectional study
Source: PLoS Med. 2022 Oct 5;19(10):e1004114. doi: 10.1371/journal.pmed.1004114 (PMC9581353; doi:10.1371/journal.pmed.1004114)
Supplement: Attachment — Submitted filename: response to editors.docx [file pmed.1004114.s007.docx]

Dear Callam and the PLOS Team,

Thank you for taking the time to review our paper again. The main request from the editors was to conduct and present an analysis that includes only the nutrition data for items with a full nutrition facts panel available, as the current approach has limited accuracy and could distort interpretation.

In our analysis, we link nutritional data on food products that have been collected from online supermarkets with brand-level sales data from the market research company Euromonitor. A ‘brand’ in our analysis can consist of multiple food products (e.g. the brand ‘Digestive biscuits’ will include sales of plain digestives, milk chocolate digestives and plain chocolate digestives). We do not have data on sales of individual products. Additionally, Euromonitor groups sales of small companies into an ‘other’ brand for each food category.

In all of our analyses, we only included products that had reported salt per 100g data – we did not impute any missing data for food products in this study. However, in the primary analysis, we did impute estimates of salt levels for the ‘other’ categories (here, salt levels were assumed to be the average of all products within all other brands from that food category). We already have a sensitivity analysis where the ‘other’ categories are removed from the analyses completely.

We have stated the total number of products that were in the original dataset and how many products were excluded due to 1) having no salt values and 2) belonging to excluded categories. This process and numbers have been added to the text of the methods (lines 140-141 and 192-194) and also to the data processing flow diagram (Fig 1). We considered any product not to have complete nutrition information if it was missing values for three other nutrients or more (kcal, carbohydrates, sugars, fat, saturates and fibre). There were only 105 products that did not meet this criteria. We excluded these and re-ran the analysis with no impact on the results. We have therefore not run this as a separate analysis, but we have stated this update in our methods and the data flow diagram. We hope this satisfies this request but are open to further discussion.

The second request was to note explicitly that nutrition data was only available for a certain number of products and that this data was extrapolated to represent a greater number of total products. This is a limitation of not using product-level sales data, which we cannot overcome. However we recognise that we have assumed that the nutrition composition data for products in this dataset are entirely representative of the brands that they matched with in the sales dataset. However, this may not be the case for several reasons: 1) any products that were out of stock on the day that the data was collected will not be included in the composition data, 2) it is possible some brands have product variants that are only available in convenience stores, markets or in restaurants and cafes, which are not captured here, and 3) products sold exclusively in-store will not be captured by this data as it was only collected online. This has been added to the discussion (lines 393-402).

The further requests below from the editors below have all been incorporated into the updated manuscript.

Please update your title to ‘Changes in the salt content of packaged foods sold in supermarkets between 2015-2020 in the United Kingdom: A repeated cross-sectional study’

The limitations mentioned by the academic editor should also be included in the final sentence of your Abstract Methods and Findings and in the Author Summary.

Data Availability Statement: Typo in first sentence (‘This’).
