# Supplementary material for: Changes in the salt content of packaged foods sold in supermarkets between 2015–2020 in the United Kingdom: A repeated cross-sectional study
Source: PLoS Med. 2022 Oct 5;19(10):e1004114. doi: 10.1371/journal.pmed.1004114 (PMC9581353; doi:10.1371/journal.pmed.1004114)
Supplement: Attachment — Submitted filename: response_to_editors.docx [file pmed.1004114.s008.docx]

Dear Callam and the PLOS Team,

Thank you for taking the time to review our paper again. The main request from the editors was to state the number of included products. While this had been done in the previous manuscript, we have rearranged the methods section to make this clearer to the reader. Please note that the content of the methods section has not changed, only the order.

The number of included products is stated in lines 161-164, and in the data processing flow chart (fig 1). No data for sodium or any other missing nutrient was imputed for any products, as stated in lines 153-154.

We have also corrected the typos that were highlighted (given below).

Lines 232/233: The word 'products' is missing.

Lines 242 and 246: Please address the typos on these lines.
